# Plant cell shape: modulators and measurements

**DOI:** 10.3389/fpls.2013.00439

**Published:** 2013-11-19

**Authors:** Alexander Ivakov, Staffan Persson

**Affiliations:** Max-Planck-Institute of Molecular Plant PhysiologyPotsdam, Germany

**Keywords:** Microtubules, actin, auxin, pavement cell, shape tools, Arabidopsis, hypocotyl

## Abstract

Plant cell shape, seen as an integrative output, is of considerable interest in various fields, such as cell wall research, cytoskeleton dynamics and biomechanics. In this review we summarize the current state of knowledge on cell shape formation in plants focusing on shape of simple cylindrical cells, as well as in complex multipolar cells such as leaf pavement cells and trichomes. We summarize established concepts as well as recent additions to the understanding of how cells construct cell walls of a given shape and the underlying processes. These processes include cell wall synthesis, activity of the actin and microtubule cytoskeletons, in particular their regulation by microtubule associated proteins, actin-related proteins, GTP'ases and their effectors, as well as the recently-elucidated roles of plant hormone signaling and vesicular membrane trafficking. We discuss some of the challenges in cell shape research with a particular emphasis on quantitative imaging and statistical analysis of shape in 2D and 3D, as well as novel developments in this area. Finally, we review recent examples of the use of novel imaging techniques and how they have contributed to our understanding of cell shape formation.

## Introduction

In immature organs where cell division is actively taking place, cells are small and the shapes of cells are largely defined by the most recent cell divisions where new cell walls have been laid down along previous division planes. The walls in such cells are straight and the cells are largely iso-diametric, bearing little resemblance to the shapes commonly observed in mature organs. To acquire their final shapes, cells must change their proportions while increasing in size. For changes in proportions to occur some parts of the cell surface must grow differently to other parts. This model of cell shape formation was proposed by Paul Green in 1965 on the basis of observations of the growth of giant internodal cells of *Nitella* and is still relevant today (Green, 1965).

In this review we aim to dissect how simple and complex cell shapes, such as those of cylindrical hypocotyl cells and leaf epidermal pavement cells, respectively, are thought to be established. A largely neglected area in the field of plant cell shape is the usage of quantitative means to define shape. We therefore also put an emphasis on how certain tools and algorithms may be used to quantify and compare shapes.

## General concepts

The shape of the cell is bounded and defined by the cell wall and it follows that cell wall expansion must be differentially regulated around the cell to generate the final cell morphology. Cell walls expand through irreversible deformation under a turgor-driven cell wall stress in a process called “creep.” Creep involves the breaking of hydrogen bonds between cell wall polymers and the reversible and irreversible breakage of covalent bonds (Cosgrove, 2005). Cell wall expansion is promoted by cell wall loosening factors, such as expansin proteins, and xyloglucan endotransglycosylases/hydrolases (XTHs) (Cosgrove, 2000; Van Sandt et al., 2007). Expansins are thought to promote cell wall creep by breaking hydrogen bonds between cellulose and xyloglucan chains (Cosgrove, 2000). XTH is able to hydrolyse covalently-bonded xyloglucan chains and re-attach them at a later time, weakening the wall (Fry et al., 1992). Cell wall stiffening may also occur. Agents proposed to stimulate cell wall stiffening include pectin methylesterase (Micheli, 2001), extensin proteins, peroxidases (Passardi et al., 2004), and reactive oxygen species (Schopfer, 1996). Creep is typically measured in isolated cell walls in an extensiometer, where the wall is loaded with a constant load and the irreversible lengthening of the wall is measured (Cosgrove, 2000).

Differential cell wall expansion underlying shape formation can include growth rates varying locally as some parts of the wall expand faster than others, as well as growth anisotropy, where the rates of expansion of a given segment of the wall are different in different directions. Anistropy is distinguished from isotropy, where the rates of expansion are equal in all directions. Cell wall anisotropy is determined by the structure of the cell wall and the arrangement of cellulose within it. Cellulose consists of high molecular weight glucan chains arranged in partially-crystalline bundles held together by numerous hydrogen bonds. These structures, termed microfibrils, have a high tensile strength and strongly resist deformation in the presence of a stretching force. The strong microfibrils are embedded in a pliant gel-like matrix consisting of hemicelluloses and pectin polysaccharides. This fiber and matrix configuration of the cell wall has been likened to a composite material (e.g., fiber-glass) and cell walls have been shown to behave mechanically like such material (Kerstens et al., 2001; Fratzl et al., 2004). The orientation of the cellulose microfibrils within the cell wall is therefore an important determinant of the behavior of the wall during growth as well as in mature tissues (Burgert and Fratzl, 2009). Microfibrils resist expansion most strongly parallel to themselves and less strongly in other directions and thus form the basis for anisotropy.

Cellulose is produced by cellulose synthase (CesA) complexes which are mobile in the plasma membrane and extrude glucan chains into the cell wall. Cellulose microfibrils have long been observed to co-align with microtubules in the cell cortex (Green, 1962). More recently, observations of fluorescently-labeled CesA complexes in the plasma membrane showed that their movement is guided by cortical microtubules (Paredez et al., 2006). Hence, by controlling microtubule organization plant cells can control the arrangements of cellulose microfibrils, and therefore the pattern of wall expansion and cell morphogenesis (Wymer and Lloyd, 1996; Lloyd, 2011). Plant microtubules are mobile and highly dynamic structures which exhibit a treadmilling movement generated by a fast rate of net polymerization at the plus end and a slower rate of depolymerisation at the minus end (Shaw et al., 2003). Microtubules interacting with each other have emergent self-organizing properties and spontaneously organize into parallel bundles or arrays (Wasteneys and Ambrose, 2009). These microtubule arrays have the ability to rapidly re-organize to new orientations. Such re-organization control CesA trajectories and microfibril arrangements, which in turn influence cell shape.

## Cylindrical cells

Anisotropic expansion has been shown to be responsible for the formation of shape in cylindrical cells, such as those of *Nitella* and *Chara*, but also of expanding root and hypocotyls cells in Arabidopsis. When cells in a multicellular organ grow in this manner, cylindrical organs such as stems, roots and hypocotyls are produced. Quantification of local strain rates in growing roots indicate a high degree of anisotropy in the expansion zone which contributes to the elongated cylindrical shape of the cells and of the entire organ and observations of microfibril orientations can largely explain the observed anisotropy (Baskin et al., 1999; Anderson et al., 2010).

### Cellulose deposition

Genetic and pharmacological experiments have shown that anisotropic expansion is greatly compromised in plants with decreased levels of cellulose (See Table S1 for an overview of mutants affecting cell shape). Cellulose-deficient mutants and plants treated with the cellulose synthesis inhibitor isoxaben exhibit radial swelling and dramatic decreases in cell elongation in hypocotyls and roots (Arioli et al., 1998; Fagard et al., 2000; Refrégier et al., 2004; Fujita et al., 2013). Presumably, in the presence of insufficient amounts of cellulose the cell wall is unable to resist the force of turgor and expands uncontrollably.

However, radial swelling is also sometimes observed in mutants with normal amounts of cellulose and with unaltered cellulose microfibril orientations, such as the temperature-sensitive microtubule organization mutant *mor1-1* (Sugimoto et al., 2003). Observations of this mutant led Wasteneys (2004) to propose the “microfibril length regulation hypothesis” (Wasteneys, 2004) whereby microtubules also control the length of deposited cellulose microfibrils. According to this hypothesis, in certain states of microtubule disruption, cellulose microfibrils are short and unable to resist the expansion of the cell wall due to insufficient hydrogen bonding with the cell wall matrix. These defects would then lead to radial swelling of the cells and decreased anisotropy.

### Microtubule organization

Various mutants with defects in microtubule organization or stability have defects in establishing anisotropic expansion and exhibit isotropic, swollen cells, as well as various degrees of helical twisting of cylindrical organs. Right-handed twisting has been observed in the *spiral1* (Furutani et al., 2000; Hashimoto, 2002; Nakajima et al., 2004; Sedbrook et al., 2004) and *spiral2/tortifolia1* mutants (Buschmann et al., 2004; Shoji et al., 2004). These mutants had altered microtubule orientations in hypocotyl cells. The products of these genes were identified as novel plant microtubule-associated proteins. When α- and β-tubulin were modified through the addition of GFP or other tags, right-handed growth was observed (Abe and Hashimoto, 2005), while left handed twisting is observed in the semi-dominant α-tubulin mutants *lefty1* and *lefty2* (Hashimoto, 2002; Thitamadee et al., 2002; Abe et al., 2004) and in the conditional microtubule organization mutant *mor-1* (Whittington et al., 2001), which exhibits severely disorganized microtubules at restrictive temperatures. Moreover, Ishida et al. (2007) examined a collection of missense and deletion mutants in α- and β-tubulin and observed both right and left-handed helical growth, depending on the position of the mutation (Ishida et al., 2007). In addition, helical twisting can be observed when microtubules are impaired with microtubule-directed drugs (Furutani et al., 2000; Nakamura et al., 2004).

It has been suggested that helical twisting and radial swelling in microtubule-related mutants may be caused by an altered pattern of CesA movement and a consequent alteration in the orientations of cellulose microfibrils in the cell wall (Wada, 2012). Helical patterns of microfibril deposition have been predicted to produce torsion in cylindrical *Nitella* cells growing anisotropically (Probine, 1963). Interestingly, right handed twisting has recently been described in the *pom2/csi1* mutant (Bringmann et al., 2012). POM2/CSI1 is a large microtubule-associated protein which also interacts with the CesA complex and was shown to be essential for the attachment of CesA complexes to microtubules. In its absence, CesA complexes lose their microtubule guidance and this leads to a partial loss of growth anisotropy and right-handed helical twisting of cylindrical organs (Bringmann et al., 2012; Li et al., 2012; Landrein et al., 2013). Closer examination of the pattern of cellulose deposition in cell walls revealed that the typical transverse cellulose orientation observed in wild type cells had given way to a tilted helical pattern (Landrein et al., 2013). The authors suggested that cellulose deposition in the absence of microtubule guidance may have a natural preponderance to occur in a curved, helical manner at an angle to the cell axis, thus producing twisted growth, and plants may use microtubule guidance of CesA complexes to counteract this tendency. Hence, microtubule organization dictates the arrangements of cellulose microfibrils, which is essential for directed growth of cylindrical cells.

### Actin cytoskeleton and cellular trafficking

In contrast to animal cells, where the microtubule cytoskeleton is primarily involved in intra-cellular transport, plant cells rely almost exclusively on the actin cytoskeleton as a transport network in interphase cells. The efficient transport of materials throughout the cell is crucial for cell growth and cell wall synthesis. Unlike cellulose, most cell wall matrix polysaccharides (with the exception of callose) are produced in the Golgi apparatus, and all plasma membrane and cell wall proteins are produced in the ER and subsequently trafficked through the Golgi apparatus, and secreted into the wall. The synthesis and the regulation of cell wall expansion thus depend on the efficient delivery of new wall material and growth-regulating factors to all cellular locations.

The importance of actin-based movement for cell growth and cell shape control is evident from drug experiments and mutants with disrupted actin networks. The resulting phenotypes, e.g., growth retardation and loss of anisotropy, are presumably caused by decreased delivery of new material to growing cellular locations (e.g., the cell wall). CesA complexes are assembled in the Golgi on their way to the plasma membrane and fluorescently-labeled CesA proteins have recently been used to probe the role of actin in cell wall synthesis. CesA-containing Golgi were seen to be less mobile upon actin depolymerisation and in the *act2act7* double mutant with impaired actin cytoskeleton, while rates of exocytosis of new CesA particles and endocytosis of CesA and FM4-64 dye were also compromised (Crowell et al., 2009; Sampathkumar et al., 2013). Drug-treated and mutant plants had cell wall defects and exhibited cellulose deficiency, accompanied by cell swelling and more isotropic growth of root cells (Gilliland et al., 2002; Kandasamy et al., 2009). Thus, the actin cytoskeleton appears to be a crucial factor in cell wall assembly and directed cell growth.

Interestingly, the *act2act7* mutant has also been shown to exhibit helical twisting (Gilliland et al., 2002; Kandasamy et al., 2009; Sampathkumar et al., 2013), a phenotype traditionally observed in microtubule-related mutants. It has recently been shown that actin filaments and microtubules transiently interact in living plant cells and a crosstalk between the actin and microtubule cytoskeleton was implicated (Sampathkumar et al., 2011). The actin cytoskeleton is further needed for re-assembly of the microtubules (Sampathkumar et al., 2011), while stabilization, and partial disruption, of actin using jasplakinolide disorganized the cortical microtubules and the movement patterns of CesAs in the plasma membrane (Sampathkumar et al., 2013). Thus, while the actin cytoskeleton may also have direct effects on cell wall synthesis independently of its transport role, it is still unclear whether this occurs through the effect of actin on microtubule organization.

## Pavement cells

**Figure 1:**
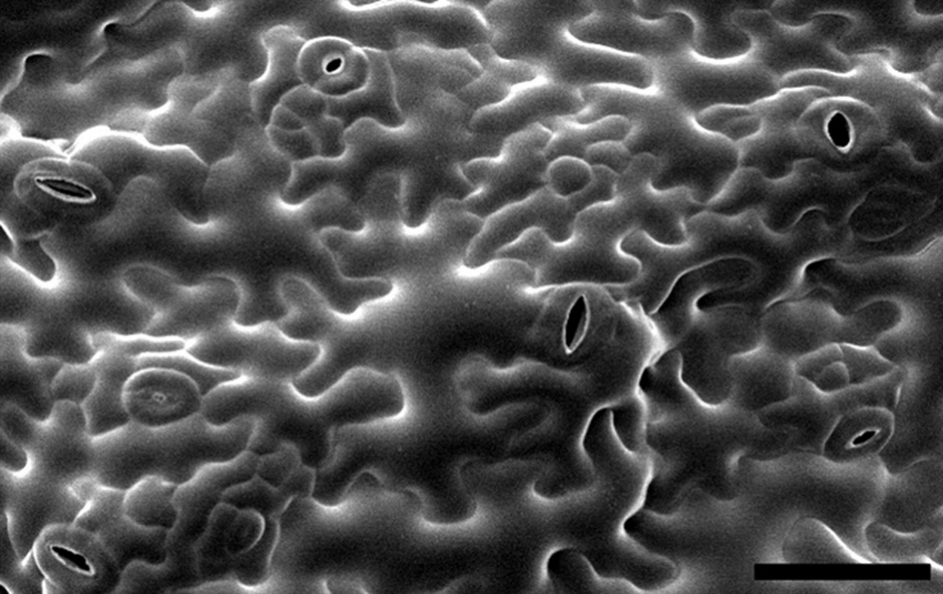
**Leaf epidermal pavement cells on the abaxial surface (underside) of an Arabidopsis cotyledon**. Image is reproduced with permission from Dolan and Langdale (2004). Scale bar: 50 μm.

Leaf epidermal pavement cells are an example of a cell type where multipolar growth patterns emerge to generate complex irregular cell shapes (Figure 1). Pavement cells have attracted much interest in recent years as a model of shape formation that goes beyond the simple scheme observed in cylindrical cells. In some species, pavement cells form a regular undulating pattern of lobes and indentations in their anticlinal cell wall which has been likened to a jigsaw puzzle.

### Cytoskeletal coordination

Just like in cylindrical cells, the cytoskeleton is thought to be central to the formation of shape in pavement cells. However, the picture seems to be more complex than in cylindrical cells, and interactions between the two cytoskeleton components appear to be required for pattern formation which establishes multi-polar growth.

**Figure 2:**
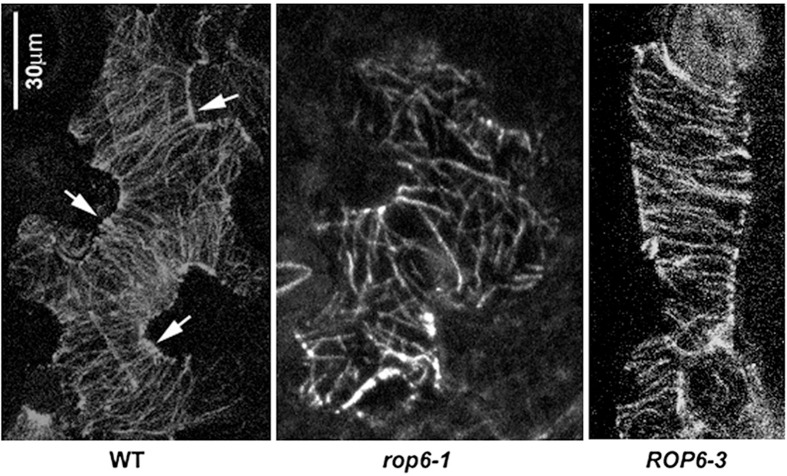
**Organization of cortical microtubules in leaf epidermal pavement cells**. Typical organization of microtubules in wild-type (left panel), *rop6-1* (mid panel) and *ROP6-3* over-expressor (right panel) leaf epidermal pavement cells visualized by an anti-tubulin antibody. Arrows indicate ordered transverse microtubules in the neck regions of wild-type cells. Image is reproduced with permission from Fu et al. (2009). Scale bar: 30 μm.

Microtubules have been observed to aggregate into parallel bundles arranged transversely across future indentation regions (Fu et al., 2005; Zhang et al., 2011), restricting local growth there, presumably through their control of the ordered deposition of cellulose microfibrils (Figure 2). Between the indentation regions, the outgrowth of lobes is associated with the aggregation of cortical fine actin filaments that are thought to promote local growth, possibly through the stimulation of vesicular trafficking and the preferential targeting of growth-promoting factors to these regions.

The regular organization of microtubules interspersed with fine actin has been shown to be generated through regulatory interactions between the cytoskeleton and the switch-like RAC/ROP-GTP'ase proteins (Fu et al., 2005, 2009). In particular ROP2 and ROP4, when in their active GTP-bound form, act redundantly to stimulate the aggregation of fine actin filaments by activating the ROP effector RIC4, while locally inhibiting the assembly of ordered microtubule arrays. Through a counteracting pathway, the ROP-interacting, microtubule-binding RIC1 protein inhibits the local assembly of fine actin where microtubules are abundant through inactivation of ROP2 and promotes microtubule assembly, thus making cortical fine actin and microtubules mutually-inhibiting and the microtubules self-activating. More recently, another pathway involving ROP6 GTP'ase was shown to promote the assembly of ordered microtubule arrays by activating the RIC1 protein, adding another piece to the puzzle (Fu et al., 2009). In such a system the two cytoskeletal components may be expected to self-organize into a pattern of mutually-exclusive local domains which then generate a regular lobed pattern through their opposing effects on local cell wall expansion. Over-activation of either pathway causes a complete loss of lobe formation as one component completely displaces the other around the entire cell periphery. This is exemplified in plants overexpressing RIC1 under the 35S promoter that causes dense transverse arrays of microtubules to form throughout the cell, resulting in block-like cells (Fu et al., 2005). This is also similar to constitutive activation of ROP2 (Fu et al., 2002, 2005) and constitutive over-expression of ROP6 (Fu et al., 2009; Lin et al., 2013). These results put an emphasis on precise coordination between the microtubule and actin cytoskeleton during complex cell shape formation.

Disruptions of the cytoskeleton and cytoskeleton-related genes further underscore its importance in pavement cell shape formation. Dual mutants of any two of the three actin protein iso-variants expressed in vegetative tissues in Arabidopsis disrupt pavement cell morphology, with the strongest reported effect in *act2act7*, where lobe formation is severely suppressed (Kandasamy et al., 2009). A number of genes encoding plant homologs of the ACTIN RELATED PROTEIN 2/3 (ARP2/3) complex have recently been identified in morphological screens with defects in cell shape formation. The ARP2/3 complex is known in other organisms as an actin modulator and appears to promote the nucleation, polymerization and cross-linking of actin and the assembly of fine cortical actin networks (Machesky and Gould, 1999). Mutants in the ARP2/3 complex genes ARP2/WURM, ARP3/DISTORTED, and ARPC5/CROOKED (Hülskamp et al., 1994; Li et al., 2003; Mathur et al., 2003a,b) all show severe defects in pavement cell shape, with a near-total absence of lobes. A mutant affecting another ARP2/3 subunit, *distorted2*, did not have a dramatic pavement cell shape phenotype, but exhibited cell adhesion defects between pavement cell lobes (El-Din El-Assal et al., 2004).

The ARP2/3 complex is known to function co-ordinately with the WAVE protein complex which appears to be required for its activation (Frank et al., 2004; Gautreau et al., 2004). Similar to ARP2/3 mutants, WAVE complex mutants exhibit cell shape abnormalities in pavement cells and trichomes. Components in this complex known to affect pavement cell shape include PIROGI/SRA1 (Basu et al., 2004), GNARLED/NAP1 (Brembu et al., 2004; Deeks et al., 2004; El-Assal et al., 2004), PIRP1 (Brembu et al., 2004), IRREGULAR TRICHOME BRANCH 1/SCAR2/DISTORTED3 (Basu et al., 2005; Zhang et al., 2005) and BRICK1 in maize (Frank and Smith, 2002) and in Arabidopsis (Djakovic et al., 2006; Le et al., 2006). In addition, several links underlying the stimulation of actin polymerization by ROP's have been identified, with the WAVE complex as a central player. The WAVE subunit PIROGI/SRA1 protein interacts with active ROP's and promotes the stimulation of ARP2/3 (Szymanski, 2005). SPIKE1, a known trichome morphogenesis modulator (Qiu et al., 2002), was recently identified as a guanine nucleotide exchange factor (GEF) which interacts with and promotes the activation of ROP's, as well as interacting with the WAVE complex subunit NAP1/GNARLED and promoting the activation of actin polymerization through the ARP2/3 complex (Basu et al., 2008).

Microtubule disruptions also severely affect pavement cell morphogenesis. The microtubule plus-end binding protein CLASP has been shown to be important for proper microtubule organization and stability (Ambrose et al., 2007; Kirik et al., 2007). In the *clasp1* mutant cortical microtubules exhibit an increased frequency of detachment from the cortex, are less dense and hyper-parallel and the pavement cell shape defective (Kirik et al., 2007; Wasteneys and Ambrose, 2009). MOR1 was identified in a screen for defective microtubule organization and the mutant exhibited severely defective pavement cell shapes, with a near-absence of lobes (Whittington et al., 2001; Kotzer and Wasteneys, 2006). The dynamic reorganization of microtubules involves targeted microtubule severing mediated by the protein katanin (Bichet et al., 2001). The katanin mutants *fra2* and *ktn1-3* have defective pavement cell shapes (Burk et al., 2001; Lin et al., 2013). Katanin was recently shown to be a downstream component of the ROP6-RIC1 pathway and was activated by its interaction with RIC1 (Hamant, 2013; Lin et al., 2013). RNAi and overexpression of MAP18, a novel microtubule associated protein proposed to destabilize microtubules, also caused defective lobe formation (Wang et al., 2007). Taken together, the organization and coordination of the cytoskeletal components are highly influential on the differential growth of a cell.

### Anisotropy

It has often been assumed that cell wall anisotropy must be involved in the generation of the shapes of pavement cells (Fu et al., 2005). However, to what extent this is true has been unclear as there has been little experimental verification. Kerstens et al. (2001) used extensiometry to show that the lobed leaf epidermis of *Kalanchoe blossfeldiana* expands isotropically in response to wall stress as a tissue. The authors used polarization confocal microscopy and determined that the tissue does not exhibit a predominant orientation of cellulose microfibrils, being essentially isotropic (Kerstens et al., 2001). This was contrasted from block-like onion epidermal cells, which had a net longitudinal orientation of cellulose microfibrils and expanded highly anisotropically, with higher expansion rates perpendicular to the orientation of cellulose microfibrils (Kerstens et al., 2001; Suslov and Verbelen, 2006; Suslov et al., 2009).

Attempts to address questions of differential growth and growth anisotropy have traditionally followed the approach of kinematic analysis (Silk and Erickson, 1979). In its simplest form it is exemplified by the classic experiment where ink dots are painted onto a root at regular intervals and their position examined over time to estimate local growth rates and directions. More recently sophisticated kinematic approaches have been applied in 2D and even 3D to infer the growth patterns of plant leaves and roots. These experiments have used exogenously-applied dots as landmarks (Poethig and Sussex, 1985; Basu et al., 2007; Remmler and Rolland-Lagan, 2012) or have tracked features present in the material being observed, such as local gray-level image structure (Schmundt et al., 1998; van der Weele et al., 2003) and mutationally-induced clonal cells which can be distinguished from their surroundings (Rolland-Lagan et al., 2003, 2005).

A result of kinematic experiments conducted on plant leaves was the surprising finding that growing dicot leaves exhibit little growth anisotropy (Erickson, 1966; Poethig and Sussex, 1985), even though local growth rates vary strongly both spatially and temporally (Poethig and Sussex, 1985; Schmundt et al., 1998). A limitation of these studies was the use of relatively mature leaves, while it is known that many developmental decisions are made early in development (Cookson et al., 2005). More recently Remmler and Rolland-Lagan (2012) tracked fluorescent micro-particles applied to the surface of young leaves to estimate local expansion rates in three dimensions and found high degrees of growth anisotropy only during short, initial stages of leaf development and largely isotropic growth for the remainder of leaf expansion (Remmler and Rolland-Lagan, 2012). The results of the experiments with leaves illustrate that varying growth rates alone may be sufficient to alter shape even in the absence of anisotropy. However, lobe formation is a cellular phenomenon and, even though the properties of individual cells may be expected to define the behavior of a tissue to some extent, local variation may cancel out over a collection of cells, particularly if it is adaptive for it to do so. Thus, the behavior of an entire tissue may not be indicative of cellular-level processes. Therefore, growth and cell wall properties must be examined at the cellular level. It is only very recently that this has been attempted and some studies are beginning to shed light on this subject.

Again, kinematic analysis is proving a useful tool, even though it is challenging to conduct kinematic analysis at the level of a cell wall in a single cell, as intra-cellular landmarks are usually non-stationary, while cell walls are largely uniform in texture and lack landmarks themselves. Zhang et al. (2011) attempted to quantify the expansion of the anticlinal cell wall in pavement cells of growing Arabidopsis cotyledons by defining the junctions between cells as landmarks and observing the cells as they expanded over a period of several days. Surprisingly, it was found that different segments of the sinusoidally-curved anticlinal wall grew at largely-equal relative rates throughout the cell, indicating isotropy. The cells also expanded essentially isometrically and changed little in shape while increasing in size, following an initial period during which lobes were initiated.

Zhang et al. (2011) pointed out that the lateral growth of pavement cells through which lobes are created cannot be explained simply by turgor-driven yielding of the anticlinal wall because there is no pressure gradient between adjacent cells. In addition, the authors speculated that the anticlinal wall may respond uniformly to tensions generated in the periclinal walls. The outer periclinal wall is subject to “tissue tension” and is strongly reinforced to resist this force (Kutschera and Niklas, 2007; Kutschera, 2008). As a “stress skin,” the outer wall is thought to provide a mechanical constraint to expansion in plant stems, thus exerting a control on the growth not only of cells, but of entire organs, forming the basis of the “epidermal growth control theory” (Kutschera and Niklas, 2007). In a similar manner, the epidermis of leaves has also been shown to limit leaf expansion (Marcotrigiano, 2010). It is thus logical that differential growth of the underlying tissue must be accompanied by differential growth in the growth-limiting outer periclinal wall. At the cellular level, anisotropic growth of this wall could generate lobes and indentations in the anticlinal walls beneath. In addition, Armor and Overall. (personal communication; University of Sydney) tracked fluorescent microparticles on growing Arabidopsis cotyledon pavement cells and concluded that the upper periclinal cell wall expands anisotropically and that this could explain lobe formation, confirming the above predictions (Armour et al., pers. Commun.).

Partial loss of anisotropy is commonly observed in cell wall-related mutants and the contribution of anisotropy to pavement cell formation can thus be assessed. Various cellulose-related mutants have been shown to exhibit simpler pavement cell morphologies with wider indentations and shorter lobes or a complete absence of lobes (Williamson et al., 2001). The *rsw2* mutant, defective in the KORRIGAN endo-1,4-β-glucanase involved in cell wall remodeling, exhibits swollen pavement cells with dramatically simplified shape (Lane et al., 2001), similar to *rsw3*, a cellulose-deficient mutant impaired in a putative glucosidase II, involved in N-glycan processing in the ER (Burn et al., 2002). More recently the *anisotropy1* mutant was identified as an allele of AtCesA1 which decreased cellulose crystallinity without affecting cellulose content and caused the loss of pavement cell lobing (Fujita et al., 2013). These data highlight the importance of ordered microfibril deposition for anisotropic growth patterns observed in complex cell shapes, such as those in pavement cells.

## Other effectors

### Hormones

#### Auxin

A relatively new, but anticipated, addition to the pavement cell puzzle is the role of plant hormones in the generation of pavement cell shapes. Various hormones are involved in almost all aspects of plant morphogenesis and may also be expected to be important in highly-polar pavement cells. From the point of view of cell shape control, auxin signaling is interesting because auxin stimulates cell expansion and has long been known to be important for the establishment of cell and organ polarity. Treatment of plants with the synthetic auxin naphthalene acetic acid (NAA) was shown to cause an increase in the frequency of anticlinal wall oscillation and the cells produce a larger number of shallow lobes (Xu et al., 2010; Li et al., 2011), while quadruple *yucca* mutants with defects in auxin biosynthesis exhibit reduced interdigitation. The latter may be rescued by supplying external auxin (Xu et al., 2010), providing the first indication that auxin may also be involved in the establishment of polarity in pavement cells.

Various auxin transporters exhibit polar localization on the plasma membrane and contribute to establishing and maintaining directional auxin flow. An example is the PIN1 auxin-efflux transporter, which localizes to the basal surface of cylindrical root stele cells, contributing to auxin flow toward the root meristem (Blilou et al., 2005). The polar targeting of PIN1 protein is strongly dependent on its phosphorylation status, where phosphorylation by PINOID kinase promotes apical localization, and de-phosphorylation by a PP2A phosphatase promotes targeting to the basal end (Friml et al., 2004; Michniewicz et al., 2007; Kleine-Vehn et al., 2009; Huang et al., 2010). PIN1 also localizes to the tips of growing lobes in pavement cells (Xu et al., 2010; Li et al., 2011). Li et al. (2011) demonstrated that the PINOID/PP2A phosphorylation switch also regulates the targeting of PIN1 in pavement cells in a strikingly similar manner to that observed in roots. Hyper-phosphorylation of PIN1 through the over-expression of PINOID, or in the knockout of the FYPP1 gene encoding a PP2A phosphatase, caused PIN1 to localize to indentations instead of lobes, suppressing lobe formation. Over-expression of PIN1 stimulated lobe formation in a similar manner to auxin treatment, while the *pin1* mutant produces pavement cells devoid of lobes (Xu et al., 2010).

It has been assumed that lobe formation in pavement cells is mediated by intra-cellular interactions between the actin and microtubule cytoskeleton, with the crosstalk mediated by ROP GTP'ase switches (Fu et al., 2005, 2009; Mathur, 2006). However, pavement cells do not develop in isolation and the outgrowth of lobes must be coordinated with the in-growth of indentations in neighboring cells. Such prefect coordination implies the existence of inter-cellular signaling between pavement cells. Auxin is again a good candidate as it is a mobile morphogen.

Significant bi-directional crosstalk between auxin signaling and RAC/ROP GTP'ases is also known (Wu et al., 2011). Xu et al. (2010) explored the connection between auxin signaling and the ROP2 and ROP6 GTP'ases involved in pavement cell formation. It was demonstrated that auxin activates ROP2 and ROP6 in protoplasts in a dose-dependent manner, as well as stimulating both the ROP2/RIC4 and the ROP6/RIC1 pathways, previously known to activate fine actin and microtubule assembly respectively. This activation was dependent on a functional AUXIN BINDING PROTEIN1, which has been proposed to function as a cell-surface auxin receptor (Badescu and Napier, 2006; Xu et al., 2010). The results taken together allowed the authors to propose auxin as a local intercellular signal coordinating lobe outgrowth with the generation of indentations in neighboring cells by stimulating the assembly of microtubule arrays across the anticlinal cell wall (Xu et al., 2010, 2011). Other recently identified mutants further strengthen the role of auxin in pavement cell development. In a yeast two-hybrid screen using constitutively-active ROP proteins as a bait the INTERACTOR OF CONSTITUTIVE ACTIVE ROPs 1 (ICR1) protein was identified (Lavy et al., 2007). The protein is a novel RAC/ROP effector which is thought to function as a scaffold for the interactions of ROP's with their interaction targets. The *icr1* and the somewhat-related *icr2* mutants exhibit a complete absence of lobes in their adaxial pavement cells. Hazak et al. (2010) subsequently showed that the polarized localization of multiple PIN proteins is impaired in the *icr1* mutant, resulting in abnormal auxin distribution and severe developmental phenotypes throughout the plant. Auxin also stimulated *ICR1* expression and ICR1 is thought to function in an auxin-regulated positive feedback loop and functions to integrate auxin signaling with ROP-based cell polarity regulation (Hazak et al., 2010). These reports thus nicely link auxin signaling to modulations of the cytoskeletal components.

#### Cytokinin

While auxin appears to promote pavement cell interdigitation by activating ROPs, cytokinin has recently been shown to act antagonistically to auxin and suppress interdigitation (Li et al., 2013). Mutants with suppressed cytokinin signaling and cytokinin receptor mutants exhibited enhanced interdigitation while over-stimulation of cytokinin signaling suppressed or abolished lobe formation. The suppression of intedigitation appears to again involve the ROPs, as activation of cytokinin signaling was shown to counteract the auxin-induced activation of ROP2 (Li et al., 2013).

### Cellular trafficking

Vesicular trafficking has long been implicated in pavement cell development. It is often assumed that cortical fine actin filaments stimulate the outgrowth of lobes by promoting the delivery of vesicles, similar to what is known in tip-growing cells such as root hairs (Yang, 2008). The entire non-cellulose component of plant cell walls is delivered through secretion, while CesA localization at the plasma membrane depends on more specialized vesicular trafficking. In addition, wall loosening agents such as expansins and XTHs must also be delivered through vesicles. More localized secretion of these agents may be involved in generating differential growth. The establishment of a bulge on Arabidopsis root hair initials is accompanied by local up-regulation of the activity of XTHs (Vissenberg et al., 2001) and expansins (Baluska et al., 2000) and such a mechanism may also be involved in the formation of pavement cell lobes, although this has not been demonstrated.

The ICR1 protein, known to be involved in pavement cell formation as discussed above, has been shown to be important for the regulation of vesicle trafficking by ROPs and the *icr1* mutant has decreased rates of exocytosis of PIN2, while endocytosis is unaffected (Hazak et al., 2010). ICR1 is also known to interact with the plant homolog of the Exocyst complex subunit Sec3 (Lavy et al., 2007), important for the tethering of secretory vesicles to the plasma membrane in preparation for fusion (Terbush et al., 1996) and the polarized targeting of exocytic events to growing buds in budding yeast (Wiederkehr et al., 2003). Active ROPs could recruit ICR1-Sec3 complexes to the plasma membrane (Lavy et al., 2007), while the *icr1* mutant had generally compromised exocytosis in root cells (Hazak et al., 2010). One of the viable mutants defective in Exocyst function, *exo84b-2* exhibits a mildly reduced complexity of pavement cell shape (Selbach, 2012). In addition, mutants in proteins of the Transport Protein Particle II (TRAPPII) complex, involved in post-Golgi vesicle trafficking and exocytosis, exhibit severe pavement cell phenotypes, with almost complete loss of lobbing, as well as defective and incomplete cell walls between cells (Qi et al., 2011).

Endocytosis appears to also be important in cell wall synthesis and modification and cell shape formation. Cell wall-derived pectin is reportedly endocytosed into endosomal compartments, perhaps for re-mobilization to other parts of the cell wall (Baluška et al., 2002). Endocytosis is also important for the regulation of cellulose synthesis by regulating CesA abundance at the plasma membrane through removal. Although it is unclear whether clathrin or a clathrin-independent mechanism is involved (Dhonukshe et al., 2007; Crowell et al., 2009), recent work suggest that one of the proteins in the adaptor complex AP2 may interact with primary wall CesA proteins *in vitro* (Bashline et al., 2013). Furthermore, the *rsw9* mutant, deficient in DYNAMIN RELATED PROTEIN 1A, has severely compromised clathrin-mediated endocytosis and exhibits cellulose deficiency, altered cell wall composition and other cell wall-related phenotypes, including cell swelling in roots (Collings et al., 2008). Furthermore, ROP2 was recently shown to promote the polar localization of PIN1 in lobe tips of pavement cells by inhibiting its endocytosis, thus adding another link to the bi-directional interactions between auxin and ROP signaling (Nagawa et al., 2012). Thus, ROP-mediated cell polarization not only affects the cytoskeleton but appears to also be crucial in the pattern forming processes determining polarized vesicular trafficking.

### Post-imaging: quantitative analysis of shape

The recent use of complex cell shapes, such as pavement cells and trichomes, as model systems for cell shape formation has increased the difficulty in describing and comparing their phenotypes. Such shapes are irregular, highly variable and exhibit a high level of complexity which is often too great to establish a clear phenotypic description in an objective, quantitative manner. Thus, methods are required to quantify and compare shapes. The broad field of morphometrics concerns itself with the quantitative analysis of shape and morphology. Numerous methods to quantify and compare shapes have been devised and are reviewed here, with a particular emphasis on their relevance to shapes commonly observed in plants.

Shape consists of geometric information. Lengths and widths, the coordinate positions of defined feature points and ratios between object dimensions are all examples of such information. Some geometric information also carries information not related to the shape itself, for example the coordinates of specific points are influenced by the shape's location and would change if the shape were moved or rotated. By its formal definition, shape is the geometric information which is independent of size, location and rotation and must thus be separated from these factors.

#### Distance methods

Distance methods are the simplest and one of the earliest forms of morphometrics. Distance methods attempt to describe a shape by measuring the distances between defined shape features, such as the lengths of limbs. Distance data is easy to collect and readily interpretable. The lengths of lobes, the widths of indentations and the lengths of trichome branches have been used to quantify the shape phenotypes of these complex cells (Fu et al., 2005, 2009; Le et al., 2006).

A chief disadvantage of such methods is their dependence on size. In plant cells, changes in shape are typically accompanied by an increase in size, however, the converse is not always true and increasing size may not be accompanied by differential growth. Thus, shape does not always change with increasing size, even in complex cells such as pavement cells. Growth of pavement cells can be isometric, as kinematic studies have shown (Zhang et al., 2011). Many mutants have defects in plant and leaf size and grow at a slower rate. Furthermore, Arabidopsis leaves do not appear to have a clear cessation of growth and continue to expand asymptotically throughout their lives (Cookson et al., 2005). It has been shown that the duration of cell and leaf expansion is also an important factor in the regulation of leaf growth, with leaves of slow-growing mutants often continuing to grow for a longer time (Cookson et al., 2005). Using measures which reflect cell size can potentially appear to pick up differences in shape which may be driven simply by capturing cells of the same shape at a different point in their development when they are simply smaller or larger.

#### Ratio methods

The problem of size dependence can be overcome by normalizing the distances to each other or to other measures of size and expressing them as ratios or proportions. Such ratios are true descriptors of shape as they are independent of size, location and rotation. The ratio of length to width is a classic shape measure, often used to describe the shapes of leaves (Tsukaya, 2003). For simple shapes, such as cylindrical cells, the length/width ratio may describe the bulk of the shape variation and is thus a very appropriate measure for their comparison. In pavement cells, the number of lobes has been normalized on cell area (Xu et al., 2010; Li et al., 2011, 2013) to obtain a quantitative indicator of interdigitation.

Other ratio-based shape descriptors attempt to compare a shape with simple reference shapes such as a circle. For this, the area of the shape is expressed as a ratio of the area of the smallest reference shape that can enclose it. These measures are easy to calculate and do not require the manual marking of shape features, such as lobes. There have been several examples of their use on pavement cells and trichomes, e.g., circularity (Le et al., 2006; Zhang et al., 2011), and new measures of this type have been devised to quantify specific aspects of cellular morphology, such as “swelling factor,” defined by comparing the area of a shape to an enclosing equilateral triangle (Basu et al., 2008), shape factor (Kirik et al., 2007) and form factor (Djakovic et al., 2006).

Distance and ratio methods suffer from their lack of uniqueness. Thus, two very differently-shaped cells may have the same circularity and will not be distinguished. A different measure may be used to distinguish these cells, however, this choice of measure is largely arbitrary, and such a measure may not always be found. Comparing the results of different studies is hampered if different measures have been used. Generally, such measures reflect a particular aspect of shape to some degree, while ignoring other aspects. These measures thus carry only limited information about a shape and may not always be informative, especially when the complexity of the shapes is high.

#### Landmark methods

Landmark morphometrics, also known as geometric morphometrics, attempts to compare shapes by comparing the coordinate positions of a set of defined common points, usually obtained by manual marking. As coordinates are typically influenced by position, rotation and size, they may first be brought into *registration* to superimpose the shapes in such a way as to make the landmark positions comparable. This is generally done by minimizing the sum of squared distances between all corresponding landmarks through scaling, translation and rotation to remove information on size, location and orientation respectively. The aggregate sum of distances between all pairs of corresponding points then represents how dissimilar the shapes are. Generalized Procrustes Analysis is a commonly-used method of achieving registration and extracting shape similarity measures from landmarks and can be applied in two, three or more dimensions (Gower, 1975; Rohlf and Slice, 1990).

Landmark methods are much used in comparative morphological studies of animals, where a variety of anatomical structures may be used to define landmarks, e.g., bones, wing structure in insects. In plants it is often difficult to define common points between structures. In leaves, the tips and bases and the junctions between veins may be used (Sinclair and Hoffmann, 2003), but this is not always applicable, e.g., in leaves which have only one tip and base. Recently MicroCT scanning was used to extract 3D models of entire orchid flowers, which were then analyzed using three-dimensional landmark methods (Van Der Niet et al., 2010). Most cell shapes in plants lack common landmarks, except a tip and base in some cases. It may be supposed that the tips of pavement cell lobes or trichome branches may be used as landmarks, however, cells differ widely in the number of lobes and trichome tips. It is also impossible to decide which lobe on one cell corresponds to a lobe on another cell, as the cells usually have no symmetry and no tip and base. Pseudolandmarks, defined by arbitrary sampling of a cell outline or surface at regular intervals, may be used in some cases and have been shown to perform well on some cell shapes (Pincus and Theriot, 2007).

#### Outline methods

The use of distance and landmark methods often requires manual measurement of shape features or marking of landmarks. Such techniques inherently suffer from bias and inaccuracy as they are influenced by skill and training level, personal preferences, fatigue and emotional and motivational factors such as experimenter's bias, as well as being time-consuming and limited in throughput. In some cases landmarks can also be difficult or impossible to identify (see above). For these reasons it may sometimes be desirable to extract and process shape information independently of landmarks. Morphometric techniques for the comparison of 2D outlines and 3D surfaces attempt to describe the entire outline or surface in the form of continuous mathematical functions and then compare the parameters of these functions, thus obviating the need to identify landmarks. Such methods typically operate on data of a low dimension (such as 2D or 3D coordinates) and transform it to a vector representation in a higher number of dimensions, thus providing a multivariate descriptor where the variables typically carry information about the entire shape, rather than specific points on it.

Examples of outline methods for continuous 2D outlines are elliptic Fourier analysis (Granlund, 1972; Kuhl and Giardina, 1982; Rohlf and Archie, 1984) and eigenshape analysis (Lohmann, 1983). The first method decomposes the shape outline as a Fourier series of trigonometric harmonic functions of increasing frequency, generating a series of Fourier coefficients which represent the shape, while the second method provides an angular representation of the outline which is then represented by a series of eigenvectors. Both methods do not strictly depend on landmark points, however, they do require digitized outline coordinates and a starting point on the outline, which may be arbitrary. The descriptors are also variant with respect to rotation and some form of registration is required. Approaches to normalize for rotational effects include Procrustes alignment of pseudolandmarks on the outline before analysis (Frieß and Baylac, 2003) and rotation of harmonics after encoding as Fourier coefficients to align the first harmonic between all shapes (Kuhl and Giardina, 1982).

The subsequent encoding in a high-dimensional morphospace provides a unique and complete representation which contains all the information which describes the shape. This is evident from the fact that the entire shape can be re-constructed and visualized from the multivariate descriptor with a desired level of precision. These methods thus allow a large amount of shape information to be collected and encoded without human participation and this permits objective, unbiased comparisons of complex shapes to be made. Even though the numerical shape representations are not readily human-interpretable, they are amenable to multivariate analysis techniques and can be used for unsupervised or supervised data mining approaches such as clustering, regression and machine learning. Such approaches have the potential to pick up patterns and differences in the data which may not be readily obvious to the human eye on the background of high shape variation, especially in complex cells like pavement cells. A number of software implementations for outline analysis are available (Slice, 1998; Hammer et al., 2001; Iwata and Ukai, 2002; Perugini, 2002).

While originally devised for computer vision shape-matching applications (Granlund, 1972; Kuhl and Giardina, 1982; Chen and Ho, 1991), the use of elliptic Fourier analysis in plant biology has been increasing in recent years, and it is becoming a popular tool for the analysis of leaf and petal shapes (Iwata and Ukai, 2002; Yoshioka et al., 2004, 2005; Andrade et al., 2008, 2010), however, its use for the analysis of plant cell shape has not been published to date.

#### 3D methods

A criticism of outline methods is their ignorance of shape detail in the interior of the outline and the lack of recognition of 3D structure. They are thus not well suited to the comparison of complex 3D organs and cells, such as flowers, trichomes and spongy mesophyll cells. To this end, landmark-free methods for 3D shapes and 2D surfaces in 3D spaces have been developed. An extension of elliptic Fourier analysis to three dimensions first encodes a stack of orthoslices of the shape using conventional elliptic Fourier analysis and then subjects the profile of each Fourier coefficient through the “depth” of the shape to another Fourier transform (Niculescu et al., 2002) or interpolates the depth profiles using spline curves (Jeong and Radke, 2007). Another recent method uses conformal geometry to represent 3D surfaces and defines several new distance measures for their comparison independently of landmarks (Boyer et al., 2011; Lipman and Daubechies, 2011). Although three-dimensional landmark-free outline and surface methods have attracted interest in the medical imaging and paleontological community, they are yet to be applied to plants.

One of the challenges in 3D shape comparison is obtaining three-dimensional geometric data of sufficient resolution and the extraction of the objects of interest from it. It has been possible to obtain confocal depth-stacks of plant tissues for some time, however, automated methods for processing such imagery have been limiting. Recent years have seen a profusion of such approaches published and the capabilites are rapidly increasing. Confocal stacks have been used to reconstruct the 3D structure of leaves (Wuyts et al., 2010, 2012) and parenchyma tissue in grape berries (Gray et al., 1999). Multi-angle image acquisition was used to reconstruct three-dimensional models of whole plant organs at cellular resolution and the temporal progression of flower development in Arabidopsis could be followed in unprecedented detail (Fernandez et al., 2010). Even more recently, three-dimensional cell segmentation was used to measure *in vivo* gene expression on a cell by cell basis by detecting the signal of fluorescent markers within cells (Federici et al., 2012).

A recent implementation, called TrichEratops, reconstructs plant surfaces in three dimensions from depth-stacks acquired on a light microscope and contains tools for the analysis of trichome patterning (Failmezger et al., 2013).

#### Morphological screening

Shape is the global output of the action of numerous cellular components and can thus be used to investigate them. Using multivariate shape data as a basis for screening allows it to be used as readout for many processes simultaneously. Untangling the effects of the various components on the readout can be achieved using data mining techniques. For example cluster analysis may group diverse shape phenotypes into separate groups which may share common causal mechanisms. This notion has been used in various approaches which have aimed to identify novel cellular components or small molecule inhibitors by using shape as an output.

Teixeira et al. (2002) used the shapes of yeast nuclei to identify novel proteins involved in nuclear architecture and gene silencing (Teixeira et al., 2002). The study used manual observation of images to identify defective phenotypes. However, another study conducted an unbiased image-based screen using automated microscopy and an image processing pipeline which automatically quantified 40 parameters in human cancer cell lines, including 10 descriptors of shape, to identify two new inhibitors of protein kinases (Tanaka et al., 2005). Morphological data was also used to identify RhoGAP/GTP'ase signaling interactions in *Drosophilla* cells (Nir et al., 2010). Both commercial and open-source automated image analysis pipelines are becoming increasingly popular. CellProfiler is a powerful open-source implementation, able to extract and analyse a large array of data from a variety of images (Carpenter et al., 2006). CellProfiler was recently used in a supervised machine learning approach where a classifier was trained by human users to automatically identify particular subtle cellular phenotypes (Jones et al., 2009). However, systematic screening approaches using objective image-derived morphological data are yet to be conducted in plants.

## Perspective

It is becoming increasingly clear that hormonal regulation, chiefly auxin and cytokinin, is an important cue for cell shape formation. This input may then impact on the activity of small GTP'ases that organize the cytoskeleton, which in turn affect the location and direction of the cell wall architecture. While some of the regulatory aspects therefore are beginning to emerge, much remains to be investigated. For example, how are hormonal gradients interacting and then being translated to recruitments of certain factors to one side of the cell but not another? In addition, the behavior of certain components within the cell is largely interpreted without considering effects from neighboring cells nor from the tissue that the cell is embedded in. Hence, it will be important to understand the behavior of the cell in these types of contexts, especially with regards to the mechanical stresses that are generated when a tissue is expanding. In a wider perspective, different plant species have different developmental programs that in the end lead to great variations in cell shape. It is anticipated that we can translate some of the knowledge gleaned in Arabidopsis to understand processes in other species, but it is currently unclear how this will play out. Lastly, integrative platforms using quantitative tools to measure cell shape variations and modeling of intracellular processes will certainly be very useful to a more complete understanding of how cells obtain their shapes.

### Conflict of interest statement

The authors declare that the research was conducted in the absence of any commercial or financial relationships that could be construed as a potential conflict of interest.
